# Morphological, hematological, biochemical characteristics, and food preferences of common teal (*Anas crecca*) during the wintering period in Punjab, Pakistan

**DOI:** 10.5455/javar.2025.l974

**Published:** 2025-12-25

**Authors:** Shozab Seemab Khan, Tariq Javed, Muhammad Saleem Khan, Muhammad Wajid

**Affiliations:** Department of Zoology, Faculty of Life Sciences, University of Okara, Okara, Pakistan

**Keywords:** Sexual dimorphism, migratory waterfowl, wintering birds, avian physiology, wetland ecology, blood profile

## Abstract

**Objective:**

The study aims to investigate sexual dimorphism in common teal (*Anas crecca*) by comparing various morphological, hematological, and biochemical parameters between males and females.

**Materials and Methods:**

27 freshly captured wintering teals were collected from hunters holding valid shooting licenses and were subjected to hematological, biochemical, and food preference analysis following all relevant ethical guidelines for animal research.

**Results:**

Several morphological traits showed significant sexual dimorphism, including body length, wingspan, primary wing length, tail length, beak length, and head length, while body weight, tarsal and metatarsal lengths, and body circumference showed no significant differences. Hematological parameters such as red blood cells count, mean corpuscular hemoglobin, mean corpuscular hemoglobin concentration, and red cell distribution width-standard deviation differed significantly between sexes. Biochemical analysis revealed notable differences in urea, protein, and albumin levels. No significant difference in dietary preferences was observed between males and females.

**Conclusion:**

Morphological differences between males and females were observed except for parameters such as body weight, tarsal length, metatarsal length, and body circumference. Dietary preference was non-significant between genders. These findings would contribute to a deeper understanding of sexual dimorphism in common teal and may inform future research on migratory behavior, habitat use, and conservation strategies tailored to sex-specific ecological needs.

## Introduction

Pakistan is home to numerous wetlands that provide a suitable habitat for a variety of migrating birds, including ducks, geese, and swans, during the winter months [1,2]. Migratory water birds comprise about one-third of the total 611 bird species that are reported from Pakistan [3]. The birds migrate from Europe and Central Asian countries to Pakistan to avoid the harsh winter [4]. Therefore, one of the seven flight routes, the Indus flyway, is present in Pakistan, and birds arrive in the Karakorum and the forests of the Suleiman [5].

**Figure 1.**
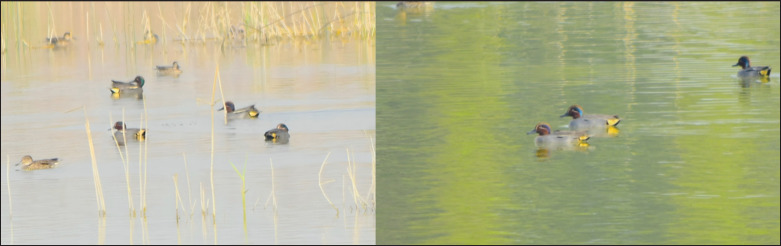
A Group of male and female common teal in Pakistan.

The common teal (*Anas crecca*) is a small migratory duck that typically resides in Western Europe but is also distributed across North Africa and much of Asia [6]. The bird breeds in the Euro-Siberian region and migrates southward during winter [7]. In Pakistan, sightings of the species have been documented across multiple wetlands (Fig. 1) [8]. The common teal occupies a wide range of wetland habitats, including both saline and freshwater environments such as rivers, lakes, estuaries, and even shallow coastal zones [9]. The species typically favors shallow waters with aquatic vegetation, especially those less than a meter deep [10].

The common teal is the smallest dabbling duck, and 34–43 cm in length and 360 gm in weight in drakes and 340 gm in hens [11]. The drakes have grey nuptial plumage from a distance, with a dark head, a yellowish back, and a white stripe running along the flanks. Eggs are placed alternately, and incubation begins when the coupling is almost complete [12]. Incubation lasts 27–28 days, and young birds start flights after 50–60 days. The ducks are precocious and can swim completely when they leave [13].

During the non-breeding season, the drake resembles the female, exhibiting a uniform coloration with a dark head and faint facial markings. The female displays a yellowish-brown plumage, slightly darker on the wings and back. Juveniles share a similar appearance to the females. Downy chicks resemble those of other dabbling ducks, being brown on the upper parts and yellow underneath, with a noticeable yellow stripe above the eye. The migration of autumn appears to begin in July and continue until October. The autumnal migration of the common teal is oriented towards the southwest, and several parallel routes are known to each other [14].

Assessing avian health can effectively be done by evaluating blood-based hematological and biochemical indicators [15]. Variations in these parameters often reflect not only localized illnesses but may also signify systemic issues or the effects of environmental contaminants [15,16]. To detect such influences, baseline data on healthy birds must be established for each annual cycle of both sexes. Hematological data are available for several species of waterfowl, but the information is limited [17-19]. For proper utilization of this information in controlled laboratory experimentation and in evaluating the health of wild-caught animals, normal values should be available for comparison. Data on hematological values of the common teal are available to a limited extent in the literature.

A review of duck hematology is presented by Elarabany [20], who published hematologic values of migratory species belonging to the Anatidae family, the Northern Shoveler (*Anas clypeata*) and Eurasian teal (*Anas crecca*), during migration season, but did not set reference values. Jax et al. [21] investigated the impact of red blood cell and white blood cell counts in bluish-green drakes.

Understanding the morphological and physiological characteristics of migratory birds is crucial for several ecological and conservation-related reasons [16,19,22]. Despite the ecological significance of these parameters, there is limited comprehensive data available on sex-specific morphological, hematological, and biochemical profiles of the common teal, particularly in the context of their wintering grounds in South Asia, including Pakistan. Most existing studies either lack sex-wise comparisons or fail to establish baseline reference values under natural migratory conditions. Therefore, the present study aimed to assess sexual dimorphism in morphological, hematological, and biochemical characteristics of common teal during the wintering period in Pakistan. Understanding these traits is essential for evaluating the birds’ health status, ecological adaptations, and energy demands; however, such sex-specific physiological data remain scarce in the region. This study contributes valuable baseline data for future ecological and conservation efforts targeting migratory waterfowl.

## Materials and Methods

### Ethical approval

The study was conducted after receiving an ethical clearance certificate from the Ethical Committee of the University of Okara, Punjab, Pakistan, under reference number UO\DOZ\2023\SSK1; date: 05-09-2023.

### Study area

Common Teal samples were collected from six wetlands/water-logged areas (District Faisalabad, District Bahawalnagar, Chashma Barrage, Taunsa Barrage, Head Marala, and Head Sulemaneki) of Punjab in collaboration of Punjab Wildlife Research Centre, Gatwala, Faisalabad (Pakistan).

Taunsa Barrage (31.31°N, 70.51°E), situated in the south-western region of Punjab (Tehsil Kot Addu, District Muzaffargarh), lies on the Indus River. Recognized as a Ramsar site, it serves as a vital wintering habitat for various waterbird species.
Chashma Barrage (32.39°N, 71.41°E), located in Tehsil Mianwali, covers around 327,000 hectares. This area is ecologically significant for migratory bird populations.
Marala Headworks (32.69°N, 74.49°E), positioned on the Chenab River in District Sialkot, encompasses a large lake area.
Head Sulemanki (29.51°N, 72.29°E) lies in the south-eastern part of Punjab, along the Sutlej River. The lake at this location is sustained by water inflows from the Chenab and Ravi rivers.
Bahawalnagar District (29.61°N, 73.09°E) comprises a network of brackish water bodies that provide essential feeding and resting habitats for migratory birds.
Faisalabad District (31.19°N, 73.6°E) is traversed by the Ravi and Chenab rivers, with multiple wetlands situated along their courses, offering ecological support to various avian species.

### Morphometric parameters

A total of 27 freshly captured adult wintering teals (15 males and 12 females) were obtained from licensed hunters during field visits conducted between November 2024 and February 2025. Sex determination was first carried out for each bird, followed by the assessment of various morphological parameters.

Body weight was taken using top-loading electronic balance (minimum count 0.01 gm).
Body length (from tip of the beak to end of tail), wing span (from one wing to another in outstretched wings).
Primary wing (from the bend of the wings to the tips of the longest primary feathers).
Tail length (from the base of the tail to the tip of the longest feathers).
Tarsal (from the shank to the base of the toes).
Meta-tarsal (from the ankle to the tip of the toes).
Body circumference (at the largest portion of the breast).
Beak length (from the tip of the beak to a set point where the feathering starts).
Head length (from the back of the skull to the tip of the bill).

### Blood sampling

27 blood samples (15 male, 12 female) were taken from the freshly captured adult common teal basilic vein in disposable 5 ml syringes (3 ml blood) equipped with a 22–25-gauge butterfly needle [1]. The syringes were conditioned with approximately 20 μl of liquid sodium heparin before collecting the sample, removing the sodium heparin through the needle and tube into the syringe’s butterfly. The collected blood was transferred to 5 ml Ethylene Diamine Tetra acetic Acid (EDTA) vacutainers with a unique sample number. The ethics of animals were assured in all measures. Blood samples were collected in two sets of vacutainers: one with EDTA (an anticoagulant used in hematology) and one without EDTA (for analysis of blood serum).

The blood samples from EDTA vacutainers were subjected to hematological analysis, including Total Leukocyte Count, Total Erythrocyte Count, Hemoglobin (HGB), Differential Leukocyte Count, and Packed Cell Volume (PCV), using an XP-100 Sysmex, Japan. Mean corpuscular volume (MCV), hematological parameters, mean corpuscular hemoglobin concentration (MCHC), and mean corpuscular hemoglobin (MCH) were taken from the values of the number of erythrocytes [23]. The serum chemistry of various blood parameters was performed using commercial diagnostic kits: total proteins through the biuret method, glucose using the enzymatic colorimetric method of glucose oxidase, serum urea using enzymatic colorimetry, the endpoint Berthelot method, and serum creatinine by the kinetic-reaction method of Jaffe [24].

### Food preferences

The gastrointestinal tracts of the same teal from which blood samples were taken (15 males, 12 females) were collected from hunters holding valid shooting licenses during the peak wintering season (December to February) to minimize seasonal variation. Each gastrointestinal tract was packed separately in polythene bags, labelled (field No., date, and sex), placed in an ice box, and transported to the Ornithological Laboratory, University of Okara, Punjab, Pakistan.

For further analysis, samples were refrigerated at 4°C until dissection. Each stomach was carefully dissected, and the contents were removed and rinsed with distilled water. The contents were passed through a standard stack of stainless-steel sieves (mesh sizes: 2.0 mm, 1.0 mm, 0.5 mm, and 0.25 mm) to separate dietary items based on size. The retained material was examined under a dissecting microscope (60x, SESYG306). Food items were sorted into categories, including seeds, insect parts, plant fragments, and other organic matter. Each item was identified to the lowest possible taxonomic level using standard taxonomic keys, using available descriptions in the literature [25] and a taxonomic key for animals [26]. Since all samples were collected within a consistent wintering period, seasonal dietary variation was minimized. This limitation is acknowledged and should be addressed in future longitudinal studies.

### Statistical analysis

The data were analyzed using standard statistical methods, including mean, standard error of the mean, and range, with IBM SPSS Statistics version 30. Differences between groups were assessed using an unpaired *t*-test at a significance level of 0.05. Pearson’s correlation coefficient was also calculated to examine relationships among various growth parameters [27].

## Results

### Morphometric analysis

Males exhibited significantly greater total body length (36.95 ± 0.26 cm) compared to females (35.71 ± 0.20 cm; *p <* 0.01), with similar patterns observed for wingspan, primary wing length, tail length, beak length, and head length (all *p <* 0.05; Table 1). While males were generally heavier than females (267.67 ± 5.18 gm *vs.* 257.09 ± 8.10 gm), this difference was not statistically significant (*p* = 0.091). No significant sexual dimorphism was observed in tarsal and metatarsal lengths or body circumference (*p *> 0.05). These findings suggest a general trend of larger size in males, which is consistent with sexual dimorphism commonly reported in waterfowl.

**Table 1.** Summary of morphometric variables in male, female adult common teal from Punjab, Pakistan.

Variable	Male ( *n* = 15)		Female ( *n* = 12)		Overall ( *n* = 27)		Sexual Dimorphism	
	Mean ± SEM	Range	Mean ± SEM	Range	Mean ± SEM	Range	*t* (paired)	*p*
Body weight (gm)	267.67 ± 5.18	235.00–338.00	257.09 ± 8.10	217.00–311.00	267.67 ± 5.18	217.00–338.00	1.76^NS^	0.091
Total length	36.95 ± 0.26	36.00–39.40	35.71 ± 0.20	34.00–36.40	36.95 ± 0.26	34.00–39.40	6.09**	0.000
Wing span	25.70 ± 0.31	24.00–28.50	24.88 ± 0.50	21.50–26.60	25.70 ± 0.31	21.50–28.50	2.42*	0.023
Primary wing	18.04 ± 0.20	17.50–19.10	17.40 ± 0.39	14.00–19.00	18.04 ± 0.20	14.00–19.10	3.13**	0.004
Tarsal length	3.28 ± 0.05	2.90–4.00	3.19 ± 0.06	3.00–3.70	3.28 ± 0.05	2.90–4.00	1.47^ NS^	0.155
Meta-tarsal length	4.34 ± 0.05	4.10–4.70	4.28 ± 0.10	3.70–5.00	4.34 ± 0.50	3.70–5.00	0.96^ NS^	0.345
Tail length	10.63 ± 0.20	8.70–12.00	10.17 ± 0.29	8.40–11.50	10.63 ± 0.20	8.40–12.00	2.11*	0.045
Body circumference	21.38 ± 0.11	20.00–22.00	21.31 ± 0.19	20.20–22.00	21.38 ± 0.11	20.00–22.00	0.53^ NS^	0.604
Beak length	4.29 ± 0.05	4.00–4.90	4.14 ± 0.08	3.70–4.50	4.29 ± 0.05	3.70–4.90	2.57*	0.017
Head length	3.22 ± 0.04	3.00–3.50	3.10 ± 0.06	2.70–3.30	3.22 ± 0.04	2.70–3.50	3.01**	0.006

SEM = standard error of mean, all lengths in cm.

Values are presented as Mean ± SEM.

* Indicates significant difference (*p* < 0.05); ** indicates highly significant difference (*p* < 0.01); NS = non-significant.

### Hematological parameters

Significant sex-based differences were evident in several hematological indices (Table 2). Males had notably higher red blood cell counts (2.90 ± 0.15 × 10^6^/µl) than females (2.46 ± 0.12 × 10^6^/µl; *p* = 0.038), along with significantly elevated HGB, MCV, MCH, and MCHC (*p <* 0.01 for all). Conversely, white blood cell (WBC), HCT, PLT, red cell distribution width-coefficient of variation (RDW-CV), and PDW did not differ significantly between sexes (*p* > 0.05). The marked hematological differences reflect sex-specific physiological and metabolic demands.

**Table 2.** Summary of hematological variables in male and female adult common teal collected from Punjab, Pakistan.

Variable	Male ( *n* = 15)		Female ( *n* = 12)		Overall ( *n* = 27)		Sexual dimorphism	
	Mean ± SEM	Range	Mean ± SEM	Range	Mean ± SEM	Range	*t* (paired)	*p*
WBC (×10³/µl)	233.50 ± 1.41	227.70–238.00	232.16 ± 2.86	217.50–246.00	232.68 ± 1.80	217.50–246.00	0.35^NS^	0.729
RBC (×10^6^/µl)	2.90 ± 0.15	2.09–3.22	2.46 ± 0.12	1.90–3.14	2.64 ± 0.11	1.90–3.22	2.26*	0.038
HGB (gm/dl)	16.83 ± 0.06	16.60–17.06	14.65 ± 0.15	13.80–15.50	15.50 ± 0.28	13.80–17.06	10.90**	0.000
HCT (%)	41.39 ± 2.63	31.00–48.40	42.45 ± 0.78	38.50–46.40	42.03 ± 1.09	31.00–48.40	-0.46^ NS^	0.649
MCV (fl)	145.37 ± 0.96	142.00–148.70	138.58 ± 1.35	131.50–145.20	141.22 ± 1.20	131.50–148.70	3.63**	0.002
MCH (pg)	66.19 ± 4.58	51.60–80.40	45.55 ± 1.06	38.40–49.50	53.58 ± 3.04	38.40–80.40	5.39**	0.000
MCHC (gm/dl)	43.99 ± 2.92	35.40–54.20	33.25 ± 0.82	28.50–38.50	37.42 ± 1.74	28.50–54.20	4.27**	0.001
PLT (×10³/µl)	7.14 ± 0.80	4.00–9.00	6.00 ± 0.52	4.00–9.00	6.44 ± 0.45	4.00–9.00	1.25^ NS^	0.228
RDW-SD (fl)	46.99 ± 2.908	38.60–57.10	31.17 ± 0.766	26.90–36.00	37.32 ± 2.206	26.90–57.10	6.39**	0.000
RDW-CV (fl)	12.31 ± 1.719	6.90–16.50	15.30 ± 0.813	8.60–17.60	14.14 ± 0.876	6.90–17.60	-1.76^ NS^	0.097
PDW (fl)	7.57 ± 0.369	6.00-9.00	7.09 ± 0.392	5.00-9.00	7.28 ± 0.278	5.00-9.00	0.84^ NS^	0.416
MPV (fl)	7.69 ± 0.321	6.70-9.00	7.03 ± 0.278	5.40-8.50	7.28 ± 0.219	5.40-9.00	1.52^ NS^	0.148
P-LCR (%)	15.86 ± 1.090	13.00-19.60	15.84 ± 0.660	12.80-18.90	15.84 ± 0.565	12.80-19.60	0.02^ NS^	0.986

Values are presented as Mean ± SEM.

* Indicates significant difference (*p* < 0.05); ** indicates highly significant difference (*p* < 0.01); NS = non-significant.

### Biochemical blood analysis

Among the biochemical markers (Table 3), males exhibited significantly higher serum total protein and albumin levels compared to females (*p <* 0.05 and *p <* 0.01, respectively). Alanine transaminase (ALT) was also significantly elevated in males (*p <* 0.001), whereas aspartate transaminase (AST), urea, and creatinine levels did not differ between sexes (*p* > 0.05). The elevated ALT and protein levels in males suggest a higher metabolic rate or liver activity relative to females.

**Table 3.** Summary of biochemical blood values variables in male and female adult common teal collected from Punjab, Pakistan.

Variable	Male ( *n* = 15)		Female ( *n* = 12)		Overall ( *n* = 27)		Sexual Dimorphism	
	Mean ± SEM	Range	Mean ± SEM	Range	Mean ± SEM	Range	*t* (paired)	*p*
Urea (mg/dl)	28.00 ± 1.77	23.00–36.00	29.73 ± 1.63	23.00–40.00	29.06 ± 1.20	40.00–17.00	−0.69^NS^	0.498
CREAT (mg/dl)	0.79 ± 0.05	0.60–0.90	0.77 ± 0.04	0.60–1.00	0.78 ± 0.03	1.00–0.40	0.21^ NS^	0.839
ALT (µl)	416.00 ± 18.36	328.00–467.00	287.45 ± 5.26	265.00–315.00	337.44 ± 16.95	467.00–202.00	8.11**	0.000
AST	884.71 ± 29.60	765.00–964.00	986.82 ± 38.82	852.00–1271.00	947.11 ± 28.42	1271.00–506.00	−1.88^ NS^	0.079
Protein	6.20 ± 0.21	5.40–6.90	5.63 ± 0.12	5.20–6.50	5.85 ± 0.12	6.90–1.70	2.61*	0.019
Albumin	2.73 ± 0.09	2.40–3.00	2.23 ± 0.09	1.80–2.70	2.42 ± 0.09	3.00–1.20	3.73**	0.002

Values are presented as Mean ± SEM.

* Indicates significant difference (*p* < 0.05); ** indicates highly significant difference (*p* < 0.01); NS = non-significant.

### Correlation between morphometry and hematology

The relationship between morphometric values with WBCs, red blood cells (RBCs), HCT, PLT, RDW-CV, PDW, MCV, and P-LCR was non-significant except for RBCs and MCV. The hemoglobin showed a significant correlation (*p* < 0.01) with total length and tarsal length, while all other morphometric parameters were not significantly related. The MCH had a significant (*p* < 0.05) correlation with total length, primary wing, and beak length, while all other morphometric parameters were non-significantly related. The MCHC had a significant (*p* < 0.05) correlation to the head length, primary wing, and beak length, while all other morphometric parameters were non-significantly related. The red cell distribution width-standard deviation (RDW-SD) showed significant (*p* < 0.05) correlation with total length, primary wing, head length, and beak length, while all other morphometric parameters showed a non-significant relation with RDW-SD (Table 4).

**Table 4.** Correlation between morphometry values and hematological parameters of common teal.

	Body weight	Total length	Wing span	Primary wing	Tarsal	Meta tarsal	Tail length	Body circum.	Beak length	Head length
WBC	0.062	0.076	0.236	0.025	0.144	−0.071	−0.271	−0.094	0.112	0.246
	0.807	0.765	0.347	0.923	0.568	0.779	0.293	0.709	0.658	0.325
RBC	0.230	0.519*	0.113	−0.029	0.244	0.254	−0.316	0.050	0.260	0.292
	0.359	0.027	0.657	0.909	0.328	0.310	0.217	0.844	0.297	0.239
HGB	0.428	0.718**	0.184	0.413	0.498*	0.179	0.144	0.110	0.371	0.466
	0.076	0.001	0.464	0.089	0.035	0.478	0.581	0.664	0.130	0.051
HCT	−0.216	−0.110	0.012	−0.120	−0.150	-0.092	−0.438	−0.193	0.185	0.114
	0.390	0.665	0.963	0.637	0.553	0.718	0.078	0.443	0.462	0.654
MCV	0.404	0.611**	0.205	0.368	0.424	0.113	−0.050	0.081	0.280	0.394
	0.097	0.007	0.415	0.133	0.080	0.656	0.848	0.750	0.261	0.106
MCH	0.442	0.476*	0.212	0.494*	0.426	0.210	0.120	0.294	0.470*	0.426
	0.066	0.046	0.399	0.037	0.078	0.402	0.646	0.237	0.049	0.078
MCHC	0.467	0.458	0.288	0.496*	0.408	0.175	0.055	0.355	0.470*	0.491*
	0.051	0.056	0.247	0.036	0.093	0.488	0.834	0.148	0.049	0.039
PLT	−0.111	0.329	−0.065	0.151	−0.025	0.056	−0.111	−0.357	−0.023	0.128
	0.660	0.183	0.798	0.550	0.923	0.824	0.672	0.146	0.928	0.612
RDW-SD	0.463	0.562*	0.309	0.534*	0.443	0.188	0.129	0.307	0.486*	0.525*
	0.053	0.015	0.212	0.022	0.065	0.455	0.621	0.216	0.041	0.025
RDW-CV	−0.281	−0.092	−0.302	−0.388	−0.218	−0.327	−0.217	−0.362	−0.299	−0.368
	0.259	0.717	0.223	0.111	0.385	0.185	0.402	0.140	0.228	0.133
PDW	0.067	0.342	0.013	0.050	0.028	−0.072	−0.251	−0.017	0.242	0.311
	0.791	0.165	0.958	0.844	0.912	0.776	0.331	0.947	0.334	0.209
MPV	0.538*	0.244	0.046	0.104	0.528*	0.166	0.232	0.285	0.058	0.191
	0.021	0.329	0.857	0.680	0.024	0.509	0.369	0.252	0.820	0.448
P-LCR	0.410	−0.066	−0.115	−0.202	0.287	0.182	−0.138	0.381	0.001	−0.152
	0.091	0.796	0.649	0.422	0.249	0.469	0.598	0.118	0.996	0.547

Upper values indicated Pearson’s correlation coefficient; lower values indicated the level of significance at 5% probability.

* Indicates significant difference (*p* < 0.05); ** indicates highly significant difference

The urea and CREAT showed a non-significant correlation except for protein with total length. The ALT showed a significant (*p* < 0.01) relation to the total length and showed a significant relation with tarsal and head length, while all other morphometric parameters showed a non-significant relation with ALT. The AST showed a significant (*p* < 0.01) relation with the primary wing, while all other morphometric parameters showed a non-significant relation with AST. The albumin showed a significant (*p* < 0.01) correlation to the total length and showed a significant relation with head length, while all other morphometric parameters showed a non-significant relation with albumin (Table 5).

**Table 5.** Correlation between morphometry values and biochemical blood values of common teal.

Parameters	Body weight	Total length	Wing span	Primary wing	Tarsal	Meta tarsal	Tail length	Body circum.	Beak length	Head length
Urea	−0.104	−0.092	−0.427	0.008	0.015	−0.209	0.267	0.096	0.051	−0.324
	0.681	0.717	0.077	0.975	0.953	0.405	0.301	0.705	0.840	0.189
CREAT	0.006	0.054	−0.220	−0.079	0.140	−0.248	0.147	−0.224	−0.154	0.025
	0.982	0.831	0.381	0.756	0.578	0.321	0.575	0.372	0.542	0.923
ALT	0.417	0.754**	0.312	0.326	0.570*	0.239	0.157	−0.071	0.137	0.477*
	0.085	0.000	0.208	0.186	0.014	0.340	0.547	0.779	0.588	0.045
AST	0.187	−0.150	−0.008	−0.624	0.060	0.409	−0.435	0.145	−0.351	−0.381
	0.458	0.552	0.976	0.006	0.812	0.092	0.081	0.565	0.153	0.119
Protein	−0.082	0.647**	0.160	0.226	−0.137	−0.098	0.065	−0.071	0.391	0.404
	0.745	0.004	0.526	0.368	0.587	0.698	0.804	0.778	0.108	0.096
Albumin	0.148	0.758**	0.330	0.374	0.208	0.107	0.033	0.008	0.369	0.470*
	0.557	0.000	0.181	0.126	0.409	0.672	0.900	0.975	0.132	0.049

Upper values indicated Pearson’s correlation coefficient; lower values indicated the level of significance at 5% probability.

* Indicates significant difference (*p* < 0.05); ** indicates highly significant difference (*p* < 0.01).

### Food preference

There is no significant difference in the frequency of food item consumption between males and females. However, males consume more algae, spermatophyta, seeds, leaves, or whole plants, and insect larvae. Females preferred to consume more Mollusca, Arachnida, adult insects, and crustaceans. Meanwhile, the frequency of algae and seeds is close between males and females, but males consume slightly more. The same applies to adult insects, where females consume slightly more. The variations in the observed frequencies of food items consumed by males and females are likely due to chance rather than a true underlying difference in their dietary preferences. The chi-square statistic (1.44) and high *p*-value (0.99) indicate that there is no significant difference in food item consumption between males and females (Table 6).

**Table 6.** Comparison of food preference between male and female common teal collected from Punjab Pakistan.

Stomach contents	Observed frequency			Expected frequency	Percentage		Chi-square
	Male *N* : (15)	Female *N* :(12)	Male	Female	Male (%)	Female (%)	
Algae	12	11	11.84	11.16	13.95	13.58	0.002
Spermatophyta	9	7	8.24	7.76	10.47	8.64	0.07
Seeds	13	11	12.36	11.64	15.12	13.58	0.03
Leaf or Plant	10	8	9.27	8.73	11.63	9.88	0.06
Mollusca	8	9	8.75	8.25	9.3	11.11	0.06
Arachnida	7	9	8.24	7.76	8.14	11.11	0.3
Insect—Larvae	11	8	9.78	9.22	12.79	9.88	0.2
Insect—Adults	10	11	10.81	10.19	11.63	13.58	0.1
Crustaceans	6	7	6.69	6.31	6.98	8.64	0.1
Total chi-square	1.44	*p*-value	0.99	Degrees of freedom	8		

Observed frequencies are the actual counts recorded during the study.

Expected frequencies represent the theoretical counts that would be observed if there were no difference between males and females in diet preference.

## Discussion

Morphological traits are increasingly utilized to infer how animal species exploit resources and define their ecological niches within communities [28]. Pettingill [29] painted and explained in detail the external measurements that were used in ornithology. Currently, many of these measurements are rarely used in books dedicated to the taxonomy of birds [30] and in field guides for several geographic areas or large groups of birds, such as shorebirds, birds of prey, and songbirds [31].

In the current study, the total body weight (gm) was in the range of 217.00–338.00 (267.67 ± 5.180), the total length (cm) 34.00–39.40 (36.95 ± 0.261), the interval (cm) 21.50–28.50 (25.70 ± 0.305), the primary wing (cm) 14.00–19.10 (18.04 ± 0.197), the total measurement of tarsal (cm) 2.90–4.00 (3.28 ± 0.052), the meta-tarsal (cm) 3.70–5.00 (4.34 ± 0.047), the total length of the tail (cm) 8.40–12.00 (10.63 ± 0.197), the body circumference (cm) 20.00–22.00 (21.38 ± 0.113), the total length of the coil (cm) 3.70–4.90 (4.29 ± 0.054), and the length of the head (cm) 2.70–3.50 (3.22 ± 0.037). The total body length, the wingspan of the primary wing, and the length of the head were very significant (*p* < 0.01); wingspan, total length of the tail, and total bone length was significantly different (*p* < 0.05); and body weight, tarsus, metatarsal, and body circumference did not differ significantly *(p* > 0.05) between male and female. According to our results, Dunning [32] and Madge [11] reported that the common teal had a length of 50–65 cm (20–26 in), of which the body is approximately two-thirds, a wingspan of 81–98 cm (32–39 in), and a weight of 0.72–1.58 kg (1.6–3.5 lb.). For standard measurements, the wing chord is from 25.7 to 30.6 cm (10.1–12.0 inches), the peak from 4.4 to 6.1 cm (1.7–2.4 inches), and the tarsus is 4 1 to 4.8 cm (1.6–1.9 inches). These morphometric parameters are a reliable indicator of habitat and food preferences. For example, Kokoszyńsk et al. [33] reported that the ability of ducks to use a certain type of food habitat depends on the length of the neck and body. Furthermore, this information can also be useful for various topics, including conservation, ecology, biology, taxonomy, and phylogeny [34]. The observed sexual dimorphism in morphometric traits, such as total length, wingspan, and head length, may reflect ecological differentiation in flight performance, foraging strategy, or migratory endurance between the sexes. These differences could provide evolutionary advantages, such as improved reproductive efficiency in males or better energy conservation in females during long-distance migration.

The second part of the study consisted of hematology of the common teal to establish a reference value for future studies. In the literature, several studies on the plasma biochemistry and hematology of wild and domesticated birds have been reported, including those by Ortizo et al. [35] and Ghanem [36]. In migratory ducks, several studies, including on mallards in the Philippines and Asian environments [37], have been reported. However, the complete hematology and biochemistry in the case of the common teal are being reported for the first time. There was no significant difference in most of the hematological and biochemical parameters when comparing male and female common teal. Plasma biochemical and hematological assessments have become essential tools in ecological studies, providing a more comprehensive view of a bird’s physiological state compared to simple condition indices, such as body weight. It is also useful to distinguish pathogenic processes [35].

The profiles of blood chemistry and hematology are often used to evaluate the physiological state of the birds. The hematological values commonly used to monitor the health status of such animals are scarce, although they have been widely described in exotic duck breeds found in various parts of the world [38]. The mean total values of RBC, PCV, HBC, and WBC in the current study were greater than 1.72 × 10^6^ mm³, 38.09%, 11.64 gm/dl, and 18.21 × 10^3^ mm³ reported by Okeudo et al. [39] for local ducks from southeastern Nigeria. These workers reported general averages of birds of different ages that were reared extensively or semi-intensively, while our birds were being hunted during migration in Pakistan. Our higher values may reflect the seasonal migration of stock. This is supported by the 3.6 × 10^6^ mm³ erythrocyte count reported by Whittow [40] for adult dabbling ducks and by the fact that the RBC, WBC, PCV, and HBC values obtained by Ola et al. [41] in adult ducks of approximately 30 weeks also compare favorably with our figures for adult ducks.

Dolka et al. [42] reported on the species and the effects of gender on the hematological parameters of birds. Khan et al. [43] observed average increases in Hb concentration in ducks from semi-active farms and lower Hb in completely enclosed (housed) ducks, which they claimed were due to various factors such as the environment, nutrition, and the system of management. An increase in hemoglobin concentration in female pheasants was also found, suggesting that increasing levels of hemoglobin compensate for the decrease in erythrocytes during the broiler period [44].

Variations in total WBC count often serve as early indicators of potentially life-threatening infections, and leukocyte quantification provides helpful information across numerous research fields. In another study, Ortizo et al. [35] documented that platelets have important roles in homeostasis and possess phagocytic functions against foreign materials. As with WBCs, platelets also form an essential part of any hematological research related to the growth of blood studies in bird physiology.

The comparison of reported values for biochemical parameters across different bird species in various studies, including ostriches, captive birds, wild seabirds, and broiler races, suggests that the biochemical parameters of birds vary by species. This means that the results and interpretation of blood and biochemical parameters in a study of birds are not correlated with those of another avian biochemical parameter [45]. Although most hematological and biochemical parameters did not show statistically significant sex-based differences, variations in protein and albumin levels—though minor—may indicate differential metabolic demands or stress responses during migration. Increased serum protein levels may indicate superior nutritional condition or an adaptive physiological response to the physical demands of migration. These findings demonstrate the value of blood chemistry as a sensitive indicator of ecological stress and physiological condition, which is essential for evaluating habitat quality and informing conservation priorities.

This study provides novel baseline data but has limitations. The sample size (*n* = 27) is relatively small and geographically restricted to birds hunted in a specific region of Punjab, Pakistan. The study also restricts its temporal coverage to a single migratory season (2024–25). These constraints may limit the generalizability of the results to the entire wintering population of common teal. Moreover, the use of hunter-collected birds may introduce sampling bias, as these individuals might not represent the full demographic or health profile of the population.

## Conclusion

This study provides valuable baseline data by comparing morphological, hematological, and biochemical parameters between male and female common teal (*Anas crecca*). While some traits, such as body weight and most hematological values, showed no significant differences, notable dimorphism was observed in traits like total length, tail length, beak length, head length, and certain blood and biochemical indicators, including RBC count, MCH, MCHC, RDW-SD, urea, protein, and albumin. These physiological differences may reflect sex-specific metabolic demands or stress responses related to migration. The absence of significant dietary differences suggests that the sexes have similar ecological roles and food preferences in the wintering habitat. Importantly, the findings contribute to a more accurate physiological and ecological profile of wintering common teal in Pakistan, which can inform targeted conservation and management efforts, such as habitat protection and wetland health monitoring during peak migration seasons. Future research should expand to include seasonal variation, larger sample sizes, and comparisons across multiple stopover sites. Longitudinal tracking of live individuals would also help assess how environmental conditions and anthropogenic pressures influence health, diet, and migration success over time.

## List of abbreviations

ALT, Alanine Transaminase; AST, Aspartate Transaminase; CREAT, Creatinine; DLC, differential leukocyte count; EDTA, Ethylene Diamine Tetra acetic Acid; HCT, Hematocrit; HGB, Hemoglobin; MCH, Mean Corpuscular Hemoglobin; MCHC, Mean Corpuscular Hemoglobin Concentration; MCV, Mean Corpuscular Volume; PVC, packed cell volume; MPV, mean platelet volume; PDW, platelet distribution width; P-LCR, platelet - large cell ratio; PLTs, platelets; RBCs, red blood cells; RDW-CV, red cell distribution width- coefficient of variation; RDW-SD, red cell distribution width-standard deviation; SEM, standard error of mean; TEC, total erythrocytes count; TLC, total leukocyte count; WBCs, white blood cells; df, degree of freedom; *p*, probability; *n*, total number; cm, centimeter; fl, femtoliter; gm/dl, grams per deciliter; µl, microliter; μm, micrometer; ml, milliliter; mg/dl, milligram per deciliter.
